# Supplementary material for: Barriers in accessing family planning services in Nepal during the COVID-19 pandemic: A qualitative study
Source: PLoS One. 2023 May 5;18(5):e0285248. doi: 10.1371/journal.pone.0285248 (PMC10162517; doi:10.1371/journal.pone.0285248)
Supplement: S1 File — (PDF) [file pone.0285248.s001.pdf]

Transcripts

RESPONDENT 1:

Interviewer: Namaste, my name is ……………. We are doing a survey to identify the barriers to access sexual and reproductive health services. This is for the study purpose and I ensure you that your identity and information provided by you will be kept confidential. Do you like to participate in the survey?

Respondent 1: Yes, sure

Interviewer: I believed the clinic representative has reached out with the basic information about the study. We will record the study and will delete the recoding once we note down the key things from the conversations. I hope that will not be problem with you. Shall I record our conversation? If you feel uncomfortable in recording that will not be a problem. I can note down the key points.

Respondent 1: Umm, Yes. Sure. Not a problem for me.

Interviewer: Can you please introduce yourself with your educational qualification and family economic status?

Respondent 1: Umm, I am 24 years' old and is a housewife. I have completed higher secondary level and belongs to the middle-income family.

Interviewer: How many children do you have?

Respondent 1: Umm, I only have one child and he is three years old.

Interviewer: Okay. I hope he is well. Do you use FP services before or is this the first time you are using the services?

Respondent 1: Umm, I have not using any modern FP methods before as I don't have child. This is the first time I am using the FP method and currently I am using short term method.

Interviewer: Okay. Great to hear. Let me talk a bit about the COVID-19 and its challenges on your community related with FP and SRH. How are you and family and how is COVID-19 in your locality?

Respondent 1: Cases are increasing day by day and the government has restricted complete lockdown. There is no movement. People are only allowed to go outside for certain time period for grocery. Schools are closed and most of the HFs are converted to COVID focused HFs.

Interviewer: Okay. Almost same in Kathmandu. We don't know when this will end. What sexual and reproductive health services do you currently seek for?

Respondent 1: Actually, I visit the health facility mostly for the sexual and reproductive health services i.e. umm family planning services. I am planning to use any short-term FP services as my husband with me most of the time in house. Despite of constraints I reached to HFs and used injectables.

Interviewer: During this epidemic, did you face any inconvenience in getting sexual and reproductive health care? Did you feel any inconvenience from your family when you came here, such as why you had to go at such a high risk? Like this is not the right time to take family planning services, what other people say when they see it, etc.

Respondent 1: Actually, yes. I was afraid to visit the HF thinking that the HF is one of the major places for contamination and transmission. *One of my newly married friends told me that she was not allowed to go to health facility for seeking FP services. When she talked with her husband for up-taking FP services, he told that she needs to take permission from mothers-in-law. When she asked her mothers-in-law, she told that newly married women with no child should not use any FP measures because this could lead to infertility.* I myself is also afraid of getting infected with COVID-19 because I heard that many carrier people do not show symptoms and can easily transmit.

Interviewer: If there is, how did you manage?

Respondent 1: Uhh, I called health service provider and share my SRH need and hesitation to visit the health facility. The HF in-charge counsel me to use face mask, maintain physical distance and keep sanitizing hand time-to-time. He also ensured me that the HF is taking all the precautionary measures amid COVD-19 and encourage me to visit HF for the SRH service.

Interviewer: Ok, did you encounter some inconveniences on the way to the service during this shutdown? Such as questioning the security guard, having to walk a long time, the risk of transmitting the disease, etc.

Respondent 1: Of course, due to closure of the public transportation I have to walk for an hour to reach to the HF. On the way I saw people were walking around without using face mask which I felt more vulnerable to C-19 transmission.

Interviewer: What problems did you face when you arrived at the service center, such as not having proper social distance, fear of service providers due to lack of security, lack of proper safety equipment such as hand washing and sanitizer; There is a difference between what you did in the past and what you do now.

Respondent 1: Well, everything was OK. But the crowd in the service center has made me afraid on getting infected with COVID-19. Actually, the day I visited HF was the immunization day which made the HF more crowded. The waiting space is small to maintain physical distancing.

Interviewer: What do you think should be done to ensure easy access to sexual and reproductive health during such epidemics in the future?

Respondent 1: I think the HF should provide all essential health services, provision of free health services during such crisis should be done.

Interviewer: Thank you for your time and response. These are of great help. Do you have any questions before we wrap up?

Respondent 1: Not a problem. Nice talking with you. Thank You

RESPONDENT 2:

Interviewer: Namaste, my name is …………… We are doing a survey to identify the barriers to access sexual and reproductive health services. This is for the study purpose and I ensure you that your identity and information provided by you will be kept confidential. Do you like to participate in the survey?

Respondent 2: Yes, sure. The clinic representative brief me about the study.

Interviewer: You just told that clinic representative has briefed you about the study. We will record the study and will delete the recoding once we note down the key points from the conversation. I hope that will not be problem with you. Shall I record our conversation? But if you feel uncomfortable in recording that will be fine as well.

Respondent 2: Sure. You can record it. I don’t have any issue.

Interviewer: Can you please introduce yourself with your educational qualification and family economic status?

Respondent 2: Umm, I am 30 years' old and is a housewife. I have completed Bachelor level and belongs to the good-income family. I am also a school teacher.

Interviewer: Do you have any children?

Respondent 2: Yes, I have two children. First one is eight years and next one is six years old.

Interviewer: Okay. I hope both of them and family members are well. Do you use FP services before or is this the first time you are using the services?

Respondent 2: Umm, I have been using FP before. I am using LARC at the moment.

Interviewer: Okay. Great to hear that. Let me talk more about the COVID-19 and its challenges on your community related with FP and SRH service utilization. How are you and family and how is COVID-19 in your locality?

Respondent 2: There noticed movement of seasonal migrants from India, which increase the risk of COVID-19 transmission. The government has imposed lockdown and all the basic services even are almost closed. You need to justify to go outside for any services.

Interviewer: What sexual and reproductive health services do you currently seek for?

Respondent 2: I am currently using the LARC. It's already has been the five years. I want to have the next method for FP. I could not visit because of this COVID-19 lockdown and other societal constraints.

Interviewer: During this epidemic, did you face any inconvenience in getting sexual and reproductive health care? Did you feel any inconvenience from your family when you came here, such as why you had to go at such a high risk? Like this is not the right time to take family planning services, what other people say when they see it, etc.

Respondent 2: Yes, my mother-in-law said that whoever goes to the HF will carry COVID-19 because health centers are the source of transmission so does not need to go to the health facility.

Interviewer: If So, How?

Respondent 2: Use of hand sanitizer, maintaining physical distance, using mask. And if available maximum use of helpline number to discuss on the health issues and solution. family member suggested to visit HF only if the HWs are using proper safety measures and when there is less crowd. Also, they suggested to get the counseling service through helpline services.

Interviewer: What were the inconveniences on the way to the service during this shutdown? Such as questioning the security guard, having to walk a long time, the risk of transmitting the disease, etc.

Respondent 2: Actually, I was afraid to get infected while meeting people, due to unavailability of transportation have to walk long distance to go to the health centers.

Interviewer: What problems did you face when you arrived at the service center, such as not having proper social distance, fear of service providers due to lack of security, lack of proper safety equipment such as hand washing and sanitizer; There is a difference between what you did in the past and what you do now.

Respondent 2: I found the health facility well adopted the COVID-19 precautionary measures such as distance is maintained, no change in the behavior of service provider was observed, hand sanitizer was available, hand washing corner was maintained. However, the premise of health center was small due to which there was less space to maintain 2 meter physical distance as recommended by WHO.

Interviewer: What do you think should be done to ensure easy access to sexual and reproductive health during such epidemics in the future?

Respondent 2: I would suggest, all the HFs should be prepared for such type of emergencies by keeping the extra stock of Personal Protective Gears and emergency medicine.

Interviewer: Thank you for your time and response. This information is of great help. Do you have any questions before we wrap up?

Respondent 1: Nothing from my side. Thank you for calling.

RESPONDENT 3:

Interviewer: Namaste, my name is ……………. We are doing a survey to identify the barriers to access sexual and reproductive health services. This is for the study purpose and I ensure you that your identity and information provided by you will be kept confidential. Do you like to participate in the survey?

Respondent 3: Yes, sure. Not a problem.

Interviewer: I believed the clinic representative has reached out with the basic information about the study. We will record the study and will delete the recoding once we note down the key things from the conversations. I hope that will not be problem with you. Shall I record our conversation? If you feel uncomfortable in recording that will not be a problem. I can note down the key points.

Respondent 3: Umm, Yes. Sure. Not a problem for me. You can record.

Interviewer: Can you please introduce yourself with your educational qualification and family economic status?

Respondent 3: I am 33 years' old and a grocery owner. I have completed higher secondary level education and belongs to the good-income family.

Interviewer: How many children do you have?

Respondent 3: Umm, I have two children. One is nine years and next is six years old.

Interviewer: Okay. I hope they are well. Do you use FP services before or is this the first time you are using the services?

Respondent 3: Umm, I have been using FP method before. I am currently using LARC method.

Interviewer: What sexual and reproductive health services do you currently seek for?

Respondent 3: I am using the LARC method and its already being more than five years. I want to consult and wish to continue the method.

Interviewer: During this epidemic, did you face any inconvenience in getting sexual and reproductive health care? Did you feel any inconvenience from your family when you came here, such as why you had to go at such a high risk? Like this is not the right time to take family planning services, what other people say when they see it, etc.

Respondent 3: People said that the SRH service is not important at this crisis situation so better to stay at home to be safe.

One of my friends was not allowed to seek the FP services by the family members telling her that the risk of transmission of COVID-19 is high if she goes to health facilities. It's better to have child then to have COVID-19 to all family members. [P.3].

Interviewer: If So, How?

Respondent 3: I responded them that SRH service is important as other health services and should be easily and continuously accessible.

I think that it's not a good idea to seek the FP services during this lock-down period as this does not have any immediate impacts like injuries, burns, etc. You can choose other traditional methods and when things come to normal and the lock-down ends, we can go for the modern contraceptives. However, my partner convinced me that use of modern contraceptive would help for the women's good reproductive health so a woman can seek modern method of FP services by taking necessary precautions for COVID-19 transmission. This has changed my decision and encouraged me to up-take modern contraceptive services. [P.3].

Interviewer: What were the inconveniences on the way to the service during this shutdown? Such as questioning the security guard, having to walk a long time, the risk of transmitting the disease, etc.

Respondent 3: *I am afraid of being infected with COVID-19. On the way also security personnel interrogated at several points regarding the movement. Upon answering that I am out for seeking sexual and reproductive health service, they use to laugh and make me feel ashamed.*

Interviewer: What problems did you face when you arrived at the service center, such as not having proper social distance, fear of service providers due to lack of security, lack of proper safety equipment such as hand washing and sanitizer; There is a difference between what you did in the past and what you do now.

Respondent 3: Everything was good and all the precautionary measures were well maintained.

Interviewer: What do you think should be done to ensure easy access to sexual and reproductive health during such epidemics in the future?

Respondent 3: Physical distancing should be regularly ensured.

My mothers-in-law is well educated and a retired government employee. She treats me like a daughter and frequently asked me regarding our plan on having child. She encouraged me to have planned baby and supported me in using FP services. However, this is not the case with most of women in our community. Women were considered shameless when they talk about the FP services openly in community and in home.

Interviewer: Thank you for your time and response. These are of great help. Do you have any questions before we wrap up?

Respondent 3: No any questions from my end. Thank you for the opportunity. Stay safe.

RESPONDENT 4:

Interviewer: Namaste, my name is …………… We are doing a survey to identify the barriers to access sexual and reproductive health services. This is for the study purpose and I ensure you that your identity and information provided by you will be kept confidential. Do you like to participate in the survey?

Respondent 4: Ok

Interviewer: I believed the clinic representative has reached out with the basic information about the study. We will record the study and will delete the recoding once we note down the key things from the conversations. I hope that will not be problem with you. Shall I record our conversation? If you feel uncomfortable in recording that will not be a problem. I can note down the key points.

Respondent 4: I don’t have problem with recording.

Interviewer: Can you please introduce yourself with your educational qualification and family economic status?

Respondent 4: I am 18 years’ old housewife. I have completed secondary level education and I am a daily wage labour. I belong to poor family. We are depend upon the daily wages for livelihood.

Interviewer: How many children do you have?

Respondent 4: Umm, I am currently married and I don’t have any child yet.

Interviewer: Okay. Do you use FP services before or is this the first time you are using the services?

Respondent 4: Umm, I was not using FP method before but as my husband is back, this is the first time I am using any FP method. Currently, I am using pills.

Interviewer: Okay. Great to hear. Let me talk a bit about the COVID-19 and its challenges on your community related with FP and SRH. How are you and family and how is COVID-19 in your locality?

Respondent 4: We are close to eastern boarder with India and there is huge flow of people from India to Nepal through our community. Cases are growing and there is lockdown. We are good. But there are cases of COVID-19 within our relatives and one of our relative died because of COVID-19.

Interviewer: What sexual and reproductive health services do you currently seek for?

Respondent 4: I has not been using any method before as my husband works in Darjeeling. He has been back and I think its time to use FP and now I am using pills.

Interviewer: During this epidemic, did you face any inconvenience in getting sexual and reproductive health care? Did you feel any inconvenience from your family when you came here, such as why you had to go at such a high risk? Like this is not the right time to take family planning services, what other people say when they see it, etc.

Respondent 4: The confusing information on COVID-19 has made me feel more uncomfortable to visit health facilities. For me, I need to get permission from family to seek FP services. Poverty has severe implication on pursuing FP/RH services.

Interviewer: If So, How?

Respondent 4: Convincing my husband that FP services is important to avoid unplanned pregnancy and for happy life.

Interviewer: What were the inconveniences on the way to the service during this shutdown? Such as questioning the security guard, having to walk a long time, the risk of transmitting the disease, etc.

Respondent 4: Security personnel interrogated where and why I am out of the house. This makes me feel uncomfortable to answer that I am going to get the SRH services which is majorly linked with services for the sexual life. No public transportation was available due to which I have to walk for an hour to reach the HF.

Interviewer: What problems did you face when you arrived at the service center, such as not having proper social distance, fear of service providers due to lack of security, lack of proper safety equipment such as hand washing and sanitizer; There is a difference between what you did in the past and what you do now.

Respondent 4: I did not faced any problems at the service center, however found that the service providers were much more alert on the COVID-19 transmission and has been giving the measures to be safe from the COVID-19 to all the visitors/patients.

I went to wash my hands at the health facility and found that there was no soap in the hand-washing corner and since everyone was busy to provide the health services no one's attention was drawn to pre-positioning of soap. [P.4].

Interviewer: What do you think should be done to ensure easy access to sexual and reproductive health during such epidemics in the future?

Respondent 4: Household visits by the service provider should be provisioned in such crisis situation to avoid the exposure of many people to the crisis, waiting area should be sanitized regularly and managed for the mass, physical distancing should be regularly practice.

Interviewer: Thank you for your time and response. These are of great help. Do you have any questions before we wrap up?

Respondent 4: Not a problem. Nice talking with you. Thank You. Stay safe.

RESPONDENT 5:

Interviewer: Namaste, my name is ……………. We are doing a survey to identify the barriers to access sexual and reproductive health services. This is for the study purpose and I ensure you that your identity and information provided by you will be kept confidential. Do you like to participate in the survey?

Respondent 5: Yes.

Interviewer: I believed the clinic representative has reached out with the basic information about the study. We will record the study and will delete the recoding once we note down the key things from the conversations. I hope that will not be problem with you. Shall I record our conversation? If you feel uncomfortable in recording that will not be a problem. I can note down the key points.

Respondent 5: Umm, Yes. Sure. I don't have problem with recording.

Interviewer: Can you please introduce yourself with your educational qualification and family economic status?

Respondent 5: I am 27 years' old and a housewife. I can only read and write without any formal education and is a daily wage labour. We belong to poor family and struggle for livelihood.

Interviewer: How many children do you have?

Respondent 5: Umm, I three children. The elder is seven years, next is five years and other is three years old.

Interviewer: Okay. They are in good health. Do you use FP services before or is this the first time you are using the services?

Respondent 5: Umm, I have not using any modern FP methods before as I don't have child. This is the first time I am using the FP method after this last child and currently I am using LARC. I went to HFs as I face some problems with LARC.

Interviewer: Okay. Let me talk a bit about the COVID-19 and its challenges on your community related with FP and SRH. How are you and family and how is COVID-19 in your locality?

Respondent 5: There is high cases of COVID-19 and there is lockdown in the municipality. All the schools, colleges are closed and people did not even invite you for work. You should get permission for going outside. Things are really tough here.

Interviewer: What sexual and reproductive health services do you currently seek for?

Respondent 5: After three children, we decide and I am using LARC services. I went to HFs as I feel some problem with LARC.

Interviewer: During this epidemic, did you face any inconvenience in getting sexual and reproductive health care? Did you feel any inconvenience from your family when you came here, such as why you had to go at such a high risk? Like this is not the right time to take family planning services, what other people say when they see it, etc.

Respondent 5: No, I did not faced any inconvenience from my family however I myself was afraid to go to health facility thinking that HF is the major source of infection and I may get infected easily.

Interviewer: If So, How?

Respondent 5: I talked with FCHV about my need of SRH service and my dilemma in visiting HF. She convinced me that the health workers are sincerely working to provide health services to people by using protective gears. I need not to worry on it and the only thing I have to do is follow all the suggested protective measures and you will be safe.

Interviewer: What were the inconveniences on the way to the service during this shutdown? Such as questioning the security guard, having to walk a long time, the risk of transmitting the disease, etc.

Respondent 5: Have to walk for more than an hour to reach to the health facility due to mobility restriction of the vehicles.

Interviewer: What problems did you face when you arrived at the service center, such as not having proper social distance, fear of service providers due to lack of security, lack of proper safety equipment such as hand washing and sanitizer; There is a difference between what you did in the past and what you do now.

Respondent 5: The waiting area is small and congested and anyone can get infected easily.

Interviewer: What do you think should be done to ensure easy access to sexual and reproductive health during such epidemics in the future?

Respondent 5: Waiting area of the HFs should be big, face mask should be provided by HFs. In case, any patient forgot to wear, all the HFs should have proper leg operated hand washing corner with the availability of sufficient water and hand washing soap. All the patients/clients should be asked to sanitize their hand at the entrance of the HF.

Interviewer: Thank you for your time and response. These are of great help. Do you have any questions before we wrap up?

Respondent 5: Thank you for calling. Bye.

RESPONDENT 6:

Interviewer: Namaste, my name is ……………. We are doing a survey to identify the barriers to access sexual and reproductive health services. This is for the study purpose and I ensure you that your identity and information provided by you will be kept confidential. Do you like to participate in the survey?

Respondent 6: Sure

Interviewer: I believed the clinic representative has reached out with the basic information about the study. We will record the study and will delete the recoding once we note down the key things from the conversations. I hope that will not be problem with you. Shall I record our conversation? If you feel uncomfortable in recording that will not be a problem. I can note down the key points.

Respondent 6: Umm, Yes. Sure. Not a problem for me

Interviewer: Can you please introduce yourself with your educational qualification and family economic status?

Respondent 6: I am 39 years' old and a housewife. I have obtained non-formal basic education. Our major source of income is agriculture. We belong to the medium income family comparing with other in the society.

Interviewer: How many children do you have?

Respondent 6: Umm, I three children and the youngest one is four years old.

Interviewer: Okay. I hope all of them are good. Do you use FP services before or is this the first time you are using the services?

Respondent 6: Umm, I have been using FP methods i.e. short-term methods to avoid unwanted pregnancy.

Interviewer: Okay. Great to hear. Let me talk a bit about the COVID-19 and its challenges on your community related with FP and SRH. How are you and family and how is COVID-19 in your locality?

Respondent 6: Cases are increasing day by day and the government has restricted complete lockdown. Not movement outside, we are engaged with agriculture stuffs and schools and HFs are mostly closed. People fear to talk with each other.

Interviewer: What sexual and reproductive health services do you currently seek for?

Respondent 6: I am using Injectables and wish to continue with the FP method. That is the reason I was visiting to HFs.

Interviewer: During this epidemic, did you face any inconvenience in getting sexual and reproductive health care? Did you feel any inconvenience from your family when you came here, such as why you had to go at such a high risk? Like this is not the right time to take family planning services, what other people say when they see it, etc.

Respondent 6: *One day I was ready to go to health facility for FP service. Meanwhile, my mothers-in-law asked me where and why I was going. I replied her that I was going to health facility for FP service, then she told me not to go during this period because everyone will know that you are using FP measures even when the people are thinking about how to get out of this COVID-19 emergency, and this will hamper our social status. [P.6].*

One of my friends was planning to come to health facility to seek FP services with me, but she denied coming to the health facility at the last moment. When I asked her the reason, she said that she could not speak to her mothers-in-law to get permission for seeking services. [P.6].

Interviewer: If So, How?

Respondent 6: Since I am friendly with my mother-in-law, I told her that seeking SRH services is a basic health need. If it is not taken timely can cause severe health complication and can lead to the more out-of-pocket expenses to cure. My mother-in-law was convinced and has told me to feel ok to seek the SRH services at any time.

Interviewer: What were the inconveniences on the way to the service during this shutdown? Such as questioning the security guard, having to walk a long time, the risk of transmitting the disease, etc.

Respondent 6: Actually, the security personnel interrogation on reason to be outside infront of other pediatrician has violated my right of confidentiality. Other obstacles were not that major to discuss as everyone is facing due to shut down of public transportation.

Interviewer: What problems did you face when you arrived at the service center, such as not having proper social distance, fear of service providers due to lack of security, lack of proper safety equipment such as hand washing and sanitizer; There is a difference between what you did in the past and what you do now.

Respondent 6: *I searched for the hand-washing corner in the health facility but did not find any such place. When I asked the support staff of the health facility, he told me that the corner has been closed because of no water supply. [P.6].*

Interviewer: What do you think should be done to ensure easy access to sexual and reproductive health during such epidemics in the future?

Respondent 6: Should focus on cleanliness of the HF, physical distancing, should be prepared with safety gears to continue service in the crisis situation.

Interviewer: Thank you for your time and response. These are of great help. Do you have any questions before we wrap up?

Respondent 6: Not a problem. Nice talking with you. Thank You. Bye.

RESPONDENT 7:

Interviewer: Namaste, my name is ……………. We are doing a survey to identify the barriers to access sexual and reproductive health services. This is for the study purpose and I ensure you that your identity and information provided by you will be kept confidential. Do you like to participate in the survey?

Respondent 7: Ok, let’s start

Interviewer: I believed the clinic representative has reached out with the basic information about the study. We will record the study and will delete the recoding once we note down the key things from the conversations. I hope that will not be problem with you. Shall I record our conversation? If you feel uncomfortable in recording that will not be a problem. I can note down the key points.

Respondent 7: Yes, I don’t have any problem with recording.

Interviewer: Can you please introduce yourself with your educational qualification and family economic status?

Respondent 7: I am 24 years' old housewife. I have completed higher secondary level education and belongs to the middle-income family. Agriculture is our major source of income.

Interviewer: How many children do you have?

Respondent 7: Umm, I already have two children. One son and next is daughter.

Interviewer: Okay. I hope he is well. Do you use FP services before or is this the first time you are using the services?

Respondent 7: Umm, since we already have two children and husband is back to home from India, we don't want to have next children and decided to use LARC.

Interviewer: Okay. Great to hear. Let me talk a bit about the COVID-19 and its challenges on your community related with FP and SRH. How are you and family and how is COVID-19 in your locality?

Respondent 7: Cases are increasing day by day and the government has restricted complete lockdown. There is no movement. People are only allowed to go outside for certain time period for grocery. Schools are closed and most of the HFs are converted to COVID focused HFs.

Interviewer: What sexual and reproductive health services do you currently seek for?

Respondent 7: Before this child, we are not using any FP method. Later, we decide to space the birth and I am currently using the pills as FP method. But, the pharmacy and HFs are even closed due to COVID-19.

Interviewer: During this epidemic, did you face any inconvenience in getting sexual and reproductive health care? Did you feel any inconvenience from your family when you came here, such as why you had to go at such a high risk? Like this is not the right time to take family planning services, what other people say when they see it, etc.

Respondent 7: Yes, while I shared that I am going to HF for IUD service my family told me that it is better to have a child than having COVID-19.

Interviewer: If So, How?

Respondent 7: I told them that, if we take proper protective measures, we cannot be infected with COVID and also getting IUD at this time can help us to avoid unplanned pregnancy. Since our first child is only one an half years, I am not prepared to have second child and want to give her my full time.

Interviewer: What were the inconveniences on the way to the service during this shutdown? Such as questioning the security guard, having to walk a long time, the risk of transmitting the disease, etc.

Respondent 7: HF was nearby so I did not feel any difficulty to reach HF.

Interviewer: What problems did you face when you arrived at the service center, such as not having proper social distance, fear of service providers due to lack of security, lack of proper safety equipment such as hand washing and sanitizer; There is a difference between what you did in the past and what you do now.

Respondent 7: Although the HF has adopted the precautionary measures against C-19, the services were satisfactory delivered. I felt that the Indoor waiting area should be avoided and is made outdoor with sufficient physical distancing.

My husband just returned from India during the lock-down, it has been one year he was in India. Since we already have two children and do not want to conceive the third one, we decided to use Implant as FP method. We have visited the nearby health center for the insertion of Implant but unfortunately, we were told that the trained health personnel have been appointed at the quarantine site and there is no one except her. Since it needs to be done by the trained health worker, we were suggested to use either condom or pills for the time being.

Interviewer: What do you think should be done to ensure easy access to sexual and reproductive health during such epidemics in the future?

Respondent 7: Since the waiting room is small, HF can make day-wise service delivery chart to avoid unnecessary gathering of people. Also, a coupon system can also be made.

Interviewer: Thank you for your time and response. These are of great help. Do you have any questions before we wrap up?

Respondent 7: Not a problem. Nice talking with you. Thank You

RESPONDENT 8:

Interviewer: Namaste, my name is ……………. We are doing a survey to identify the barriers to access sexual and reproductive health services. This is for the study purpose and I ensure you that your identity and information provided by you will be kept confidential. Do you like to participate in the survey?

Respondent 8: Yes, sure

Interviewer: I believed the clinic representative has reached out with the basic information about the study. We will record the study and will delete the recoding once we note down the key things from the conversations. I hope that will not be problem with you. Shall I record our conversation? If you feel uncomfortable in recording that will not be a problem. I can note down the key points.

Respondent 8: You can record. Not a problem for me.

Interviewer: Can you please introduce yourself with your educational qualification and family economic status?

Respondent 8: I am 25 years' old Dalit housewife women. I have completed my primary level education and belongs to the poor family. I am a daily wage labour.

Interviewer: How many children do you have?

Respondent 8: Umm, I three children. Two daughters and one son.

Interviewer: Okay. I hope he is well. Do you use FP services before or is this the first time you are using the services?

Respondent 8: Umm, I am using injectables as means of FP.

Interviewer: Okay. Great to hear. Let me talk a bit about the COVID-19 and its challenges on your community related with FP and SRH. How are you and family and how is COVID-19 in your locality?

Respondent 8: COVID-19 has been increasing and people afraid to meet each other. Many services have been closed and people are getting back to village from different locations.

Interviewer: What sexual and reproductive health services do you currently seek for?

Respondent 8: I was having the Injectables and its already a three month. The lockdown has restricted the movement. I went HFs to continue my FP method.

Interviewer: During his epidemic, did you face any inconvenience in getting sexual and reproductive health care? Did you feel any inconvenience from your family when you came here, such as why you had to go at such a high risk? Like this is not the right time to take family planning services, what other people say when they see it, etc.

Respondent 8: Actually, my mother-in-law was not happy to see me going to the health facility when she knew that I am going to receive family planning service. My husband is labour migrant and is returning home. We already had three children and had poor economic status, we do not want more child.

I lived in a community where male members from majority of the household go to India as seasonal migrant for living. One of my friends whose husband is also a labor migrant had recently returned home during lockdown. To have safe sexual life, she expressed her wish to have FP services but unfortunately could not go for services. When I asked her the reason for not going to clinic for FP service despite of having interest, she told me that she had to do calculated expenses for basic needs, as there is uncertainty in opening lockdown. Despite the service is free, women like her cannot afford transportation cost to reach to the clinic.

Interviewer: If So, How?

Respondent 8: I told her that giving birth to more child is easy but giving them good education, health, life is a most important. We being a parent should be responsible for the better health and education of them. She after listening this being emotional and allowed me to go to the health facility.

Interviewer: What were the inconveniences on the way to the service during this shutdown? Such as questioning the security guard, having to walk a long time, the risk of transmitting the disease, etc.

Respondent 8: Actually, on the way I saw many people are walking without wearing mask properly. Some are wearing on their chin, some are just covering their mouth, some are taking out their mask while sneezing. This behavior of people made me feel more vulnerable to COVID infection.

Interviewer: What problems did you face when you arrived at the service center, such as not having proper social distance, fear of service providers due to lack of security, lack of proper safety equipment such as hand washing and sanitizer; There is a difference between what you did in the past and what you do now.

Respondent 8: Since this was the day of child immunization, there was a crowd at the health facility making riskier for the COVID transmission.

Interviewer: What do you think should be done to ensure easy access to sexual and reproductive health during such epidemics in the future?

Respondent 8: The day for all the basic health services should be properly planned with the good emergency services.

Interviewer: Thank you for your time and response. These are of great help. Do you have any questions before we wrap up?

Respondent 8: Thank you for calling. Nice talking with you.

RESPONDENT 9:

Interviewer: Namaste, my name is ……………. We are doing a survey to identify the barriers to access sexual and reproductive health services. This is for the study purpose and I ensure you that your identity and information provided by you will be kept confidential. Do you like to participate in the survey?

Respondent 9: Yes, I am ready.

Interviewer: I believed the clinic representative has reached out with the basic information about the study. We will record the study and will delete the recoding once we note down the key things from the conversations. I hope that will not be problem with you. Shall I record our conversation? If you feel uncomfortable in recording that will not be a problem. I can note down the key points.

Respondent 9: Umm, Yes. Sure. Not a problem for me.

Interviewer: Can you please introduce yourself with your educational qualification and family economic status?

Respondent 9: I am 35 years' old and a business woman. I have completed Master's degree and belongs to the good-income family.

Interviewer: How many children do you have?

Respondent 9: Umm, I only two children- one son and one daughter.

Interviewer: Okay. I hope he is well. Do you use FP services before or is this the first time you are using the services?

Respondent 9: I have been using FP methods from before. Currently, I am using pills (short term method) for FP.

Interviewer: Okay. Great to hear. Let me talk a bit about the COVID-19 and its challenges on your community related with FP and SRH. How are you and family and how is COVID-19 in your locality?

Respondent 9: COVID-19 impact all the sectors and the cases are mostly populated in Kathmandu valley. There is a threat of going outside and even HFs are occupied with COVID-19 cases. Days are really tough.

Interviewer: What sexual and reproductive health services do you currently seek for?

Respondent 9: I have been to HFs to continue my FP methods and do some SRH check-ups.

Interviewer: During this epidemic, did you face any inconvenience in getting sexual and reproductive health care? Did you feel any inconvenience from your family when you came here, such as why you had to go at such a high risk? Like this is not the right time to take family planning services, what other people say when they see it, etc.

Respondent 9: I did not faced any inconvenience from my family but COVID-19 information such as risk of death; lack of treatment; asymptomatic transmission made me feel uncomfortable to go to health facility.

Interviewer: If So, How?

Respondent 9: I had a health worker as my neighbor, I talked with him about this dilemma and he counsel me with the right information of COVID-19.

Interviewer: What were the inconveniences on the way to the service during this shutdown? Such as questioning the security guard, having to walk a long time, the risk of transmitting the disease, etc.

Respondent 9: Interrogation by the security personnel at various point made uncomfortable. Some personnel even made joke on my reason i.e., sexual and reproductive health services of going to the health facility.

Interviewer: What problems did you face when you arrived at the service center, such as not having proper social distance, fear of service providers due to lack of

security, lack of proper safety equipment such as hand washing and sanitizer; There is a difference between what you did in the past and what you do now.

Respondent 9: HF has arranged hand washing facilities with water and soap but I did not feel ok to use the soap which is touched by many hands.

Interviewer: What do you think should be done to ensure easy access to sexual and reproductive health during such epidemics in the future?

Respondent 9: HF should maintain leg operated hand washing station and liquid soap to avoid touching by many hands.

Interviewer: Thank you for your time and response. These are of great help. Do you have any questions before we wrap up?

Respondent 9: It was good to talk with you in the pandemic. Good luck with research.

RESPONDENT 10:

Interviewer: Namaste, my name is ……………. We are doing a survey to identify the barriers to access sexual and reproductive health services. This is for the study purpose and I ensure you that your identity and information provided by you will be kept confidential. Do you like to participate in the survey?

Respondent 10: Yes,

Interviewer: I believed the clinic representative has reached out with the basic information about the study. We will record the study and will delete the recoding once we note down the key things from the conversations. I hope that will not be problem with you. Shall I record our conversation? If you feel uncomfortable in recording that will not be a problem. I can note down the key points.

Respondent 10: Umm, Yes. Sure. Not a problem for me.

Interviewer: Can you please introduce yourself with your educational qualification and family economic status?

Respondent 10: I am 29 years' old and a housewife. I have completed my bachelor and currently employed. Working in private bank.

Interviewer: How many children do you have?

Respondent 10: Umm, I two children and both of them were daughters.

Interviewer: Okay. I hope he is well. Do you use FP services before or is this the first time you are using the services?

Respondent 10: Umm, we have not using any modern FP methods before. This is the first time I am using the FP method and currently I am using short term method.

Interviewer: Okay. Great to hear. Let me talk a bit about the COVID-19 and its challenges on your community related with FP and SRH. How are you and family and how is COVID-19 in your locality?

Respondent 10: Cases are increasing day by day and the government has restricted complete lockdown. There is no movement. People are only allowed to go outside for certain time period for grocery. Schools are closed and most of the HFs are converted to COVID focused HFs.

Interviewer: What sexual and reproductive health services do you currently seek for?

Respondent 10: Initially we decide to have LARC, but we ends up with having short-term method because of unavailability of HWs for LARC.

Interviewer: During this epidemic, did you face any inconvenience in getting sexual and reproductive health care? Did you feel any inconvenience from your family when you came here, such as why you had to go at such a high risk? Like this is not the right time to take family planning services, what other people say when they see it, etc.

Respondent 10: *I belong to the economically strong family, however when I told my husband that I need to go to health facility for FP services, he opposed me for seeking services. He said that it's better to have one more child whom we can afford than COVID-19.*

Interviewer: If So, How?

Respondent 10: I made him take advice with the health worker who is his friend also. After talking with him, my husband is convinced on seeking services and go for the planned baby.

Interviewer: What were the inconveniences on the way to the service during this shutdown? Such as questioning the security guard, having to walk a long time, the risk of transmitting the disease, etc.

Respondent 10: Need to answer many times to the security personnel some also passed unnecessary comment on visiting HF for sexual and reproductive health service.

Interviewer: What problems did you face when you arrived at the service center, such as not having proper social distance, fear of service providers due to lack of security, lack of proper safety equipment such as hand washing and sanitizer; There is a difference between what you did in the past and what you do now.

Respondent 10: *I decided with my husband to use long acting reversable FP methods (IUCD) and went to health facility to seek services. However, I was unable to receive the desired services because of unavailability of required commodity i.e., IUCD set for insertion. We then, decided to use other short-term FP methods.*

Interviewer: What do you think should be done to ensure easy access to sexual and reproductive health during such epidemics in the future?

Respondent 10: The facility should also ensure the availability of FP services in all emergency situation.

Interviewer: Thank you for your time and response. These are of great help. Do you have any questions before we wrap up?

Respondent 10: Thank You. Take care. Bye.

RESPONDENT 11:

Interviewer: Namaste, my name is ……………. We are doing a survey to identify the barriers to access sexual and reproductive health services. This is for the study purpose and I ensure you that your identity and information provided by you will be kept confidential. Do you like to participate in the survey?

Respondent 11: Yes, sure

Interviewer: I believed the clinic representative has reached out with the basic information about the study. We will record the study and will delete the recoding once we note down the key things from the conversations. I hope that will not be problem with you. Shall I record our conversation? If you feel uncomfortable in recording that will not be a problem. I can note down the key points.

Respondent 11: Umm, Yes. Sure. Not a problem for me.

Interviewer: Can you please introduce yourself with your educational qualification and family economic status?

Respondent 11: I am 37 years' old and a housewife. I have completed secondary level education and belongs to the middle-income family.

Interviewer: How many children do you have?

Respondent 11: Umm, I have two children. One Son and one daughter. The youngest one is daughter and is four years old.

Interviewer: Okay. I hope he is well. Do you use FP services before or is this the first time you are using the services?

Respondent 11: Yes. After the birth of my son, I am continuing using the short term method i.e. I am using Depo (injectable) for FP.

Interviewer: Okay. Great to hear. Let me talk a bit about the COVID-19 and its challenges on your community related with FP and SRH. How are you and family and how is COVID-19 in your locality?

Respondent 11: Cases are increasing day to day and there is a news on shortage of beds and oxygen. People are afraid of going to HFs. We are good but we too are afraid because of COVID-19. All sectors have been impacted.

Interviewer: What sexual and reproductive health services do you currently seek for?

Respondent 11: I went to HFs for continuing my FP methods as it's already more than three months. We wait for one month believing that COVID-19 will ends and we can go for HFs. But, with its uncertainty, we finally decide to come and up-take it.

Interviewer: During this epidemic, did you face any inconvenience in getting sexual and reproductive health care? Did you feel any inconvenience from your

family when you came here, such as why you had to go at such a high risk? Like this is not the right time to take family planning services, what other people say when they see it, etc.

Respondent 11: For me it is not easy and comfortable to talk with my in-laws about sexual and reproductive health services. The SRH in our community is stigmatized and mostly linked with sex.

Interviewer: If So, How?

Respondent 11: I had shared about the infection with my mother-in-law and its complication if not treated timely.

Interviewer: What were the inconveniences on the way to the service during this shutdown? Such as questioning the security guard, having to walk a long time, the risk of transmitting the disease, etc.

Respondent 11: Due to shut down of public transportation, I had to walk long distance alone.

Interviewer: What problems did you face when you arrived at the service center, such as not having proper social distance, fear of service providers due to lack of security, lack of proper safety equipment such as hand washing and sanitizer; There is a difference between what you did in the past and what you do now.

Respondent 11: At the health center, there skilled health worker was deployed to the place with high COVID-19 case load.

Interviewer: What do you think should be done to ensure easy access to sexual and reproductive health during such epidemics in the future?

Respondent 11: Each health facility should make of roster of trained and skilled human resource so that all the health services will be continuously provided to the patient.

Interviewer: Thank you for your time and response. These are of great help. Do you have any questions before we wrap up?

Respondent 11: Nice talking with you. Take care. Bye.

RESPONDENT 12:

Interviewer: Namaste, my name is ……………. We are doing a survey to identify the barriers to access sexual and reproductive health services. This is for the study purpose and I ensure you that your identity and information provided by you will be kept confidential. Do you like to participate in the survey?

Respondent 12: Sure

Interviewer: I believed the clinic representative has reached out with the basic information about the study. We will record the study and will delete the recoding once we note down the key things from the conversations. I hope that will not be problem with you. Shall I record our conversation? If you feel uncomfortable in recording that will not be a problem. I can note down the key points.

Respondent 12: Sure. I don't have problem with recording.

Interviewer: Can you please introduce yourself with your educational qualification and family economic status?

Respondent 12: I am 19 years' old and a housewife. I have completed higher secondary level education and belongs to the middle-income family and is currently married (just five to six months ago).

Interviewer: How many children do you have?

Respondent 12: Umm, I am currently married just five to six months before. We don't have any child now.

Interviewer: Okay. Do you use FP services before or is this the first time you are using the services?

Respondent 12: Umm, in fact, we were not using any FP method before. This is the first time we used FP method. I am using the depo (injectables) as FP methods for the first time.

Interviewer: Okay. Great to hear. Let me talk a bit about the COVID-19 and its challenges on your community related with FP and SRH. How are you and family and how is COVID-19 in your locality?

Respondent 12: There has been restriction on the movement and most of the schools and HFs are closed either because HWs are tested positive with COVID-19 or unavailability of equipment. COVID-19 cases are increasing day by day and there also noticed deaths because of COVID-19 in community.

Interviewer: What sexual and reproductive health services do you currently seek for?

Respondent 12: Since I just get married, we are planning for not having birth for at least two years, I am now having depo.

Interviewer: During this epidemic, did you face any inconvenience in getting sexual and reproductive health care? Did you feel any inconvenience from your family when you came here, such as why you had to go at such a high risk? Like this is not the right time to take family planning services, what other people say when they see it, etc.

Respondent 12: *I told my husband that I must go to the health facility for up-taking depo (Injectables) since it has already been three months that I have injected and needs to continue. My husband was quite offensive initially arguing that the FP is not the current priority when everyone is struggling with COVID-19 situation. But when I told him that some of the nearby health facilities are offering the FP services with necessary precautions and continuation of Depo will help in our sexual and reproductive health, he was then convinced and we both went to the health facility for the FP service.*

Interviewer: If So, How?

Respondent 12: I told my husband that the HF is sincerely taking all the precautionary measures against COVID-19 and they are even using PPE gown for the protection of COVID transmission.

Interviewer: What were the inconveniences on the way to the service during this shutdown? Such as questioning the security guard, having to walk a long time, the risk of transmitting the disease, etc.

Respondent 12: Transportation was not allowed so I have to walk long distance to reach to health facility.

Interviewer: What problems did you face when you arrived at the service center, such as not having proper social distance, fear of service providers due to lack of security, lack of proper safety equipment such as hand washing and sanitizer; There is a difference between what you did in the past and what you do now.

Respondent 12: *I was in the health facility for FP services on the day of immunization. There were more than 50 people with their children in the health*

facility who came for immunization services. The waiting space at health facility was small so it was impossible to maintain the physical distancing. I really feel unsecured being in crowd in the health facility.

Interviewer: What do you think should be done to ensure easy access to sexual and reproductive health during such epidemics in the future?

Respondent 12: I think HF should have made day wise provision of basic health services with the provision of emergency services.

Interviewer: Thank you for your time and response. These are of great help. Do you have any questions before we wrap up?

Respondent 12: Thank you for your time as well.

RESPONDENT 13:

Interviewer: Namaste, my name is ……………. We are doing a survey to identify the barriers to access sexual and reproductive health services. This is for the study purpose and I ensure you that your identity and information provided by you will be kept confidential. Do you like to participate in the survey?

Respondent 13: Yes, sure

Interviewer: I believed the clinic representative has reached out with the basic information about the study. We will record the study and will delete the recoding once we note down the key things from the conversations. I hope that will not be problem with you. Shall I record our conversation? If you feel uncomfortable in recording that will not be a problem. I can note down the key points.

Respondent 13: Umm, Yes. Sure. Not a problem.

Interviewer: Can you please introduce yourself with your educational qualification and family economic status?

Respondent 13: I am 28 years' old and an employed woman. I have completed Bachelor degree and belongs to the good-income family.

Interviewer: How many children do you have?

Respondent 13: Umm, I have two children and both of them are son.

Interviewer: Okay. I hope they are well. Do you use FP services before or is this the first time you are using the services?

Respondent 13: Umm, I am using FP methods before and quit for second child. After second child, I am having the FP services.

Interviewer: Okay. Great to hear. Let me talk a bit about the COVID-19 and its challenges on your community related with FP and SRH. How are you and family and how is COVID-19 in your locality?

Respondent 13: COVID-19 increases day by day and there is increased different message relating COVID-19, which further threaten people. Movement has been restricted, shops and other are being closed. Life is really tough because of COVID-19.

Interviewer: What sexual and reproductive health services do you currently seek for?

Respondent 13: I am having the implant and I need to change it.

Interviewer: During this epidemic, did you face any inconvenience in getting sexual and reproductive health care? Did you feel any inconvenience from your family when you came here, such as why you had to go at such a high risk? Like this is not the right time to take family planning services, what other people say when they see it, etc.

Respondent 13: Actually, I and my family has no any issue on taking SRH service during pandemic.

Interviewer: If So, How?

Respondent 13: My family is well sensitized on the sexual and reproductive health problems and I need not to convince them.

Interviewer: What were the inconveniences on the way to the service during this shutdown? Such as questioning the security guard, having to walk a long time, the risk of transmitting the disease, etc.

Respondent 13: *I was going to the health facilities for seeking FP services. During my way to health facility, I saw people not using any protective measures (even mask). Every time I come across them; I feel that I might get infected with COVID-19; which is said to be highly infectious and make me feel vulnerable and unsecured.*

Interviewer: What problems did you face when you arrived at the service center, such as not having proper social distance, fear of service providers due to lack of security, lack of proper safety equipment such as hand washing and sanitizer; There is a difference between what you did in the past and what you do now.

Respondent 13: The waiting room of the health center is small and it was difficult to maintain proper physical distancing. I stayed outside of it and has entered when the crowd became less.

Interviewer: What do you think should be done to ensure easy access to sexual and reproductive health during such epidemics in the future?

Respondent 13: At such pandemic situation, the health center should arrange token system to avoid unnecessary gathering of people.

Interviewer: Thank you for your time and response. These are of great help. Do you have any questions before we wrap up?

Respondent 13: No any questions. Bye.

RESPONDENT 14:

Interviewer: Namaste, my name is ……………. We are doing a survey to identify the barriers to access sexual and reproductive health services. This is for the study purpose and I ensure you that your identity and information provided by you will be kept confidential. Do you like to participate in the survey?

Respondent 14: Sure

Interviewer: I believed the clinic representative has reached out with the basic information about the study. We will record the study and will delete the recoding once we note down the key things from the conversations. I hope that will not be problem with you. Shall I record our conversation? If you feel uncomfortable in recording that will not be a problem. I can note down the key points.

Respondent 14: Sure. No problem for recording.

Interviewer: Can you please introduce yourself with your educational qualification and family economic status?

Respondent 14: I am 24 years' old. I have completed primary level education and belongs to the poor income family. I am a daily wage labour.

Interviewer: How many children do you have?

Respondent 14: Umm, I two children. My youngest child is daughter and she is two years old.

Interviewer: Okay. I hope he is well. Do you use FP services before or is this the first time you are using the services?

Respondent 14: Umm, I have not using any modern FP methods before as I don't have child. This is the first time I am using the FP method and currently I am using short term method.

Interviewer: Okay. Great to hear. Let me talk a bit about the COVID-19 and its challenges on your community related with FP and SRH. How are you and family and how is COVID-19 in your locality?

Respondent 14: Cases are increasing day by day and the government has restricted complete lockdown. There is no movement. People are only allowed to go outside for certain time period for grocery. Schools are closed and most of the HFs are converted to COVID focused HFs. People are getting back to village from different cities and foreign countries as well.

Interviewer: What sexual and reproductive health services do you currently seek for?

Respondent 14: I went to health facilities for Depo (injectables) as this need to be within three months. I am already late by a month because of COVID-19.

Interviewer: During this epidemic, did you face any inconvenience in getting sexual and reproductive health care? Did you feel any inconvenience from your family when you came here, such as why you had to go at such a high risk? Like this is not the right time to take family planning services, what other people say when they see it, etc.

Respondent 14: I, and my husband lives here with our 2 year's old daughter. My husband told me not to go to health facility rather it's better to conceive a child. Going to the HF will increase your's and our family's risk to COVID infection.

Interviewer: If So, How?

Respondent 14: I convinced him by telling that I will take all the precautionary measures against COVID infection. One of my friend who has visited HF just two

days ago also told me that, health workers are wearing PPE gown, face shield, globes properly.

Interviewer: What were the inconveniences on the way to the service during this shutdown? Such as questioning the security guard, having to walk a long time, the risk of transmitting the disease, etc.

Respondent 14: I have to answer the reason why I am out and where I am going to the security personnel at several points on my way to health facility. This several point interrogation made me feel awkward.

Interviewer: What problems did you face when you arrived at the service center, such as not having proper social distance, fear of service providers due to lack of security, lack of proper safety equipment such as hand washing and sanitizer; There is a difference between what you did in the past and what you do now.

Respondent 14: At the health center, I saw that there was a hand washing station but there is availability of bar soap only. This has made me touch the contaminated bar which made me feel anxious.

Interviewer: What do you think should be done to ensure easy access to sexual and reproductive health during such epidemics in the future?

Respondent 14: The health center should replace the bar soap with liquid soap and should arrange leg operated hand washing tap to avoid the risk of exposure of patient.

Interviewer: Thank you for your time and response. These are of great help. Do you have any questions before we wrap up?

Respondent 14: Not a problem. Nice talking with you. Thank You. Bye.

RESPONDENT 15:

Interviewer: Namaste, my name is ……………. We are doing a survey to identify the barriers to access sexual and reproductive health services. This is for the study purpose and I ensure you that your identity and information provided by you will be kept confidential. Do you like to participate in the survey?

Respondent 15: OK

Interviewer: I believed the clinic representative has reached out with the basic information about the study. We will record the study and will delete the recoding once we note down the key things from the conversations. I hope that will not be problem with you. Shall I record our conversation? If you feel uncomfortable in recording that will not be a problem. I can note down the key points.

Respondent 15: Umm, Yes. We can proceed.

Interviewer: Can you please introduce yourself with your educational qualification and family economic status?

Respondent 15: I am 34 years' old housewife women. I have completed higher secondary level education and belongs to the middle-income family.

Interviewer: How many children do you have?

Respondent 15: Umm, I have two children. Youngest one is son and is of five years.

Interviewer: Okay. I hope he is well. Do you use FP services before or is this the first time you are using the services?

Respondent 15: Umm, I have been using FP method before the first child. I quit in between and I am using after this birth.

Interviewer: Okay. Great to hear. Let me talk a bit about the COVID-19 and its challenges on your community related with FP and SRH. How are you and family and how is COVID-19 in your locality?

Respondent 15: There has been movement restriction, school closure and increase cases of COVID-19. Health facilities are occupied with COVID-19 cases and people even afraid of visiting HFs.

Interviewer: What sexual and reproductive health services do you currently seek for?

Respondent 15: I have visited to the health facility for IUCD (long acting FP services).

Interviewer: During this epidemic, did you face any inconvenience in getting sexual and reproductive health care? Did you feel any inconvenience from your family when you came here, such as why you had to go at such a high risk? Like

this is not the right time to take family planning services, what other people say when they see it, etc.

Respondent 15: *Initially, I thought that it is safer to stay at home without using any FP measures rather than increasing exposure to COVID-19 by visiting health facility. However, later realized that the long-term impact of not using contraceptive measure will be more risky than COVID-19 and convinced myself for the services before talking with any family members including partner. My key learning from this was one should have strong will and should be prepared oneself for up taking these services during this sort of emergencies.*

Interviewer: If So, How?

Respondent 15: I talked with Female Community Health Volunteer (FCHV) on my dilemma.

Interviewer: What were the inconveniences on the way to the service during this shutdown? Such as questioning the security guard, having to walk a long time, the risk of transmitting the disease, etc.

Respondent 15: On the way to health center, I saw that people are not using their face mask properly. Every time, I pass these pediatricians made me feel highly at risk of getting infected with COVID-19.

Interviewer: What problems did you face when you arrived at the service center, such as not having proper social distance, fear of service providers due to lack of security, lack of proper safety equipment such as hand washing and sanitizer; There is a difference between what you did in the past and what you do now.

Respondent 15: At the health center, I found that the trained service provider was deployed to the quarantine center and I need to return without receiving the service.

Interviewer: What do you think should be done to ensure easy access to sexual and reproductive health during such epidemics in the future?

Respondent 15: All the health facility should have maintained sufficient number of trained HR to continue its services even in the emergency situation like today. Also, the cost of the specialized SRH service should be reduced as many of the people has lost their job due to the pandemic.

Interviewer: Thank you for your time and response. These are of great help. Do you have any questions before we wrap up?

Respondent 15: I don't have any questions. Thank you for calling.

RESPONDENT 16:

Interviewer: Namaste, my name is ……………. We are doing a survey to identify the barriers to access sexual and reproductive health services. This is for the study purpose and I ensure you that your identity and information provided by you will be kept confidential. Do you like to participate in the survey?

Respondent 16: OK

Interviewer: I believed the clinic representative has reached out with the basic information about the study. We will record the study and will delete the recoding once we note down the key things from the conversations. I hope that will not be problem with you. Shall I record our conversation? If you feel uncomfortable in recording that will not be a problem. I can note down the key points.

Respondent 16: Umm, Yes. Sure. Not a problem for me.

Interviewer: Can you please introduce yourself with your educational qualification and family economic status?

Respondent 16: I am 23 years' old housewife women. I have completed higher secondary level education and belongs to the poor family. Our major source of income is agriculture.

Interviewer: How many children do you have?

Respondent 16: Umm, I already have three children.

Interviewer: Okay. I hope they are well. Do you use FP services before or is this the first time you are using the services?

Respondent 16: Umm, I have not using any FP method. I get married in 16/17 years and already have three children. I am using FP method for the first time.

Interviewer: Okay. Great to hear. Let me talk a bit about the COVID-19 and its challenges on your community related with FP and SRH. How are you and family and how is COVID-19 in your locality?

Respondent 16: Movement has been restricted and it is really tough for us to survive as there is low income generation opportunity. No one is offering any works even in agriculture with fear of transmission of COVID-19.

Interviewer: What sexual and reproductive health services do you currently seek for?

Respondent 16: I am using Pills as per the suggestions from the Health workers.

Interviewer: During this epidemic, did you face any inconvenience in getting sexual and reproductive health care? Did you feel any inconvenience from your family when you came here, such as why you had to go at such a high risk? Like this is not the right time to take family planning services, what other people say when they see it, etc.

Respondent 16: Actually, there is a belief that FP measures have health side-effects and also lead to infertility so its better not to be the user of it. My family also belief the same.

Interviewer: If So, How?

Respondent 16: I told them that this is a misbelief and family planning service help us to plan our healthy and happy family. I also has requested our neighbour who is health worker by profession to share right information on it.

Interviewer: What were the inconveniences on the way to the service during this shutdown? Such as questioning the security guard, having to walk a long time, the risk of transmitting the disease, etc.

Respondent 16: *Very limited health facilities are in operation during this lock-down period and to those who are giving services have limited health care services to the emergency care only and the public vehicles were not allowed to operate. We need to walk for more than usual time to go to health facilities and seek services. This is the one of the major obstacles we faced during lock-down. [P.16]. When I go for services, most of the community people can notice me due to very few movements in the road. When they saw me going for services; they ask me many questions as where and why are you going, what is the urgency to visit the health facility at this emergency time, did you told your husband about your movement, and many more that affect my decision to go health facility. Also, I need to answer the security*

personnel several times. I think I cannot enjoy my right of seeking confidential FP services in such cases.

Interviewer: What problems did you face when you arrived at the service center, such as not having proper social distance, fear of service providers due to lack of security, lack of proper safety equipment such as hand washing and sanitizer; There is a difference between what you did in the past and what you do now.

Respondent 16: The waiting room of the health center was not large enough to maintain physical distance.

Interviewer: What do you think should be done to ensure easy access to sexual and reproductive health during such epidemics in the future?

Respondent 16: The health center should arrange for the specialized SRH services once in a week. The public and private health center should collaborate to ensure the regular availability of SRH services.

Interviewer: Thank you for your time and response. These are of great help. Do you have any questions before we wrap up?

Respondent 16: Thank you for your time as well.

RESPONDENT 17:

Interviewer: Namaste, my name is ……………. We are doing a survey to identify the barriers to access sexual and reproductive health services. This is for the study purpose and I ensure you that your identity and information provided by you will be kept confidential. Do you like to participate in the survey?

Respondent 17: Yes, sure

Interviewer: I believed the clinic representative has reached out with the basic information about the study. We will record the study and will delete the recoding once we note down the key things from the conversations. I hope that will not be problem with you. Shall I record our conversation? If you feel uncomfortable in recording that will not be a problem. I can note down the key points.

Respondent 17: Umm, Yes. Sure. I don’t have problem with recording.

Interviewer: Can you please introduce yourself with your educational qualification and family economic status?

Respondent 17: I am 32 years' old and a housewife. I have completed primary level education and belongs to the middle-income family.

Interviewer: How many children do you have?

Respondent 17: Umm, I have three children. The youngest one is six years old.

Interviewer: Okay. I hope they are good. Do you use FP services before or is this the first time you are using the services?

Respondent 17: Umm, I am using FP before but short term. But after this last child, I am using LARC (IUCD) for FP. I wish to continue and went HFs for the service.

Interviewer: Okay. Great to hear. Let me talk a bit about the COVID-19 and its challenges on your community related with FP and SRH. How are you and family and how is COVID-19 in your locality?

Respondent 17: Cases are increasing day by day and the government has restricted complete lockdown. There is no movement. People are only allowed to go outside for certain time period for grocery. Schools are closed and most of the HFs are converted to COVID focused HFs.

Interviewer: During this epidemic, did you face any inconvenience in getting sexual and reproductive health care? Did you feel any inconvenience from your family when you came here, such as why you had to go at such a high risk? Like this is not the right time to take family planning services, what other people say when they see it, etc.

Respondent 17: *One of my nearby friends who is a housewife says that she wishes to up-take the family planning measures from the nearest clinic but cannot discuss to her family members because they avoid any outside movement due to fear of COVID-19 transmission. She also told that the family members never consult her while making any decisions and she needs to get permission from her in-laws while going outside the home.*

Interviewer: If So, How?

Respondent 17: I have told her to request local Female Community Health Volunteers to counsel her family on the importance of family planning methods and also on allowing her to decide her sexual and reproductive health.

Interviewer: What were the inconveniences on the way to the service during this shutdown? Such as questioning the security guard, having to walk a long time, the risk of transmitting the disease, etc.

Respondent 17: Due to restriction on movement of public vehicle, I have to walk long distance. Also, on the way I got interrogated by security personnel in front of other people which I feel that there is violation of my right of privacy which is very important in a country where sexual and reproductive health is taken as a private matter.

Interviewer: What problems did you face when you arrived at the service center, such as not having proper social distance, fear of service providers due to lack of security, lack of proper safety equipment such as hand washing and sanitizer; There is a difference between what you did in the past and what you do now.

Respondent 17: *I do not notice any differences in delivering services than usual days. Only the difference I felt was that the health workers were using the personal protective equipment (apron, goggles, mask and gloves) that they usually did not use in normal days before.*

Interviewer: What do you think should be done to ensure easy access to sexual and reproductive health during such epidemics in the future?

Respondent 17: The cost of the specialized SRH services should be reduced.

Interviewer: Thank you for your time and response. These are of great help. Do you have any questions before we wrap up?

Respondent 17: No any questions. Thank You.

RESPONDENT 18:

Interviewer: Namaste, my name is ……………. We are doing a survey to identify the barriers to access sexual and reproductive health services. This is for the study purpose and I ensure you that your identity and information provided by you will be kept confidential. Do you like to participate in the survey?

Respondent 18: Yes, sure

Interviewer: I believed the clinic representative has reached out with the basic information about the study. We will record the study and will delete the recoding once we note down the key things from the conversations. I hope that will not be problem with you. Shall I record our conversation? If you feel uncomfortable in recording that will not be a problem. I can note down the key points.

Respondent 18: Umm, Yes. Sure. Not a problem for me.

Interviewer: Can you please introduce yourself with your educational qualification and family economic status?

Respondent 18: I am 24 years' old and a housewife. I have completed secondary level education and belongs to the middle-income family. Agriculture is our major source of income.

Interviewer: How many children do you have?

Respondent 18: Umm, I only two children. Both are son.

Interviewer: Okay. They are good, I believed. Do you use FP services before or is this the first time you are using the services?

Respondent 18: Umm, I have not using any modern FP methods before. After having two children, we decided to use the FP method for the first time. We are planning to use before but because of COVID-19 lockdown, we just visited for the services.

Interviewer: Okay. Great to hear. Let me talk a bit about the COVID-19 and its challenges on your community related with FP and SRH. How are you and family and how is COVID-19 in your locality?

Respondent 18: There is increase in cases and HFs are highly occupied with COVID-19. It was heard that HWs are mostly affected with COVID-19 and security personnel are patrolling. Life is tough at the moment.

Interviewer: What sexual and reproductive health services do you currently seek for?

Respondent 18: We decided to have short term method and up on suggestions from HWs we used injectable.

Interviewer: During this epidemic, did you face any inconvenience in getting sexual and reproductive health care? Did you feel any inconvenience from your

family when you came here, such as why you had to go at such a high risk? Like this is not the right time to take family planning services, what other people say when they see it, etc.

Respondent 18: *The information on transmission, fatality and stigma related to COVID-19 on different social medias like Facebook, YouTube makes me to decide on not seeking FP services during this pandemic. However, phone counseling from the health worker helps me to build the confidence for seeking services. Sometimes, the right message at right times would be great help in seeking the services during this sort of emergencies.*

Interviewer: If So, How?

Respondent 18: I have called on the toll-free helpline number and discussed my concern on seeking FP service. The health worker convinced me by clarifying the misinformation on COVID-19 transmission.

Interviewer: What were the inconveniences on the way to the service during this shutdown? Such as questioning the security guard, having to walk a long time, the risk of transmitting the disease, etc.

Respondent 18: Well, *on my way to health facility from home, which almost took 45 minutes, I was asked by the security personnel three times on my way about my movement. When I told them that I was going for seeking FP services; one of them commented as "Can't you control your sexual desire till lock-down". I really feel ashamed listening to him.*

Interviewer: What problems did you face when you arrived at the service center, such as not having proper social distance, fear of service providers due to lack of security, lack of proper safety equipment such as hand washing and sanitizer; There is a difference between what you did in the past and what you do now.

Respondent 18: *The good thing I noticed during my visit to health facility was health service providers were providing extensive counseling on COVID-19, its sign and symptoms and the preventive measures. They were also requesting the clients to report to concern authorities if someone in the community were suspected for COVID-19.*

Interviewer: What do you think should be done to ensure easy access to sexual and reproductive health during such epidemics in the future?

Respondent 18: FCHVs should be mobilized in an extensive way to provide SRH, helpline information and distribution of condom and pills.

Interviewer: Thank you for your time and response. These are of great help. Do you have any questions before we wrap up?

Respondent 18: Thank you for calling. Nothing from my side.
