# Supplementary material for: Barriers in accessing family planning services in Nepal during the COVID-19 pandemic: A qualitative study
Source: PLoS One. 2023 May 5;18(5):e0285248. doi: 10.1371/journal.pone.0285248 (PMC10162517; doi:10.1371/journal.pone.0285248)
Supplement: Attachment — Submitted filename: Response to Reviewers.pdf [file pone.0285248.s002.pdf]

Rebuttal letter

We thank the academic editor and the two reviewers for their comments on our manuscript. Below is our response to each point raised by the academic editor and reviewers. We hope that we satisfyingly addressed them and that the manuscript will be now suited for publication.

Sincerely,

On behalf of all authors,

Anil Sigdel

Academic editor:

1.Please ensure that your manuscript meets PLOS ONE's style requirements, including those for file naming.

File naming was edited to comply with the style requirements. We hopefully have no

divergences from the style requirements now.

2. In your Data Availability statement, you have not specified where the minimal data set underlying the results described in your manuscript can be found. PLOS defines a study's minimal data set as the underlying data used to reach the conclusions drawn in the manuscript and any additional data required to replicate the reported study findings in their entirety. All PLOS journals require that the minimal data set be made fully available. For more information about our data policy, please see http://journals.plos.org/plosone/s/data-availability.

Upon re-submitting your revised manuscript, please upload your study's minimal underlying data set as either Supporting Information files or to a stable, public repository and include the relevant URLs, DOIs, or accession numbers within your revised cover letter. For a list of acceptable repositories, please see http://journals.plos.org/plosone/s/data-availability#loc-recommended-repositories. Any potentially identifying patient information must be fully anonymized.

Data has been uploaded as supporting documents in the revised submission.

Reviewer #1

Thank you for letting me review this paper on barriers to family planning during the Covid19 pandemic. Overall, the study is well-conducted and address an important research

question. However, I think the manuscript can be improved, mainly to have a clearer focus on the special circumstances of Covid19 and also when it comes to general recommendations that are not bound to the Covid19 situation. Both these concerns are addressed in the paper, but could be sharpened.

Thank you for the suggestion. We updated the recommendations considering with COVID-19. Please refer the revised manuscript (clean version) discussion section (line #542-563) and conclusion section (line #565-591).

When I first read the abstract and the start of the Results section, I get the impression that this paper is about barriers to family planning in general. I think the relation to the lockdown and the pandemic can be made clearer throughout. Studies on general barriers to FP are plentifold and would not justify yet another study, but the special circumstances of a pandemic might. Especially if we can draw general conclusions on how to act in health emergencies.

We revise the conclusion section with more focus on how to act in health emergencies. Please refer the revised manuscript (cleaned version) conclusion section (line #565-591).

In line 96, page 6, you state that "interviews explored the barriers to FP". What about the special circumstances of the pandemic? How where they addressed? Please align with the aim of the study stated on lines 91-92.

The sentences have been revised and few sentences were added that will highlights about the special circumstances of pandemic and the measures we ensured to address the gaps. Please refer the line #97-105 in revised manuscript (cleaned version).

This last point, that general insights can be drawn is important, since we are not likely to see an exact copy of the lock-down situation even if there will surely be more pandemics coming. On this note, I lack an overarching theme in the Results section. You have applied the socio-ecological framework, which is good, but what is the overarching lesson learnt from this study?

Overarching lesson learnt section has been added in the result section in the revised manuscript. Please refer the line #426-437 in revised manuscript (cleaned version).

Line 117 has a broken reference

Thank you for flagging. Corrected.

Line 111 mis-spelled PI

Thank you for flagging. Corrected in the respective line.

Line 115 Saturation was reached how

We noticed that the information was saturated because the last three interviews did not add any new information complementing to the objectives and scope of the study. This has been added in line #129-131 in revised manuscript (cleaned version).

I lack a section on reflexivity in the Discussion section

We added section on reflexivity on discussion part. Please refer the line #543-553 of revised manuscript (cleaned version).

I also lack methodological considerations. What about making telephone interviews, how may that have affected results?

Why we did telephone interview was explained in line # 99-102. We highlight some impact of telephone interview in line #554-564 (last paragraph of discussion section).

Reviewer #2

Thank you for the opportunity to review this manuscript. Overall, it requires a significant edit to ensure meaning and content can be understood. This includes consistency and clarity with acronym use and a review of unclear phrasing (for eg, 'havoc messages', 'unsecured feelings' 'people living of HIV' etc.). Referencing also needs to be checked and updated throughout the document. Some references are incomplete or contain errors.

Thank you for the suggestion. Use of acronyms has been made uniform throughout the manuscript and operation definition has been added of some of the phrasing. References have been checked and updated as suggested.

Introduction

The WHO declared the COVID-19 outbreak a public health emergency of international concern on the 30th Jan 2020. A worldwide pandemic was declared March 11th 2020. Please correct this error.

Thank you for correction. Correction has been made. Please refer the line #52 in revised manuscript (Cleaned version).

The cure rate and case fatality rate provided are not helpful data in the context of this study. More contextually informative data about the COVID-19 pandemic in Nepal is required. For example, number of confirmed cases, cases/million population, confirmed deaths, deaths/million population etc. A comment on the availability of COVID-19 testing or the reliability of national COVID-19 data in Nepal is also needed. If possible and available from the Nepal government, it would be helpful to also include COVID-19 data or context specifically from the 5/77 districts in Nepal that the authors purposely selected the 18 participants in their study.

The data as suggested has been added in the revised manuscript. Please refer the line #55-59 (in revised cleaned manuscript) which highlight the national level and study sites data from government source.

Reference 3 is not useful for readers to get an overall sense of the COVID-19 pandemic in Nepal, including at the time of data collection (Jan 15th – 25th 2021). You could consider

referencing a global database such as:https://www.worldometers.info/coronavirus/country/nepal/

Thank you for the suggestion. Worldometers references has been added and data updated from the source.

A summary of the SRHR context in Nepal is needed here or in the study setting section below, with focus on access to contraception. For example, CPR, unmet need for contraception, contraceptive method-mix etc. Have previous studies looked into access barriers before the pandemic? If yes, what are they?

Thank you for the suggestion. This has been updated in study setting section. Please refer the line# 115-119 where different statistics in Nepal has been presented.

Materials and methods

Please consider including data about phone ownership and access for women in Nepal.

This has been updated in study setting section. Please refer the line #115-119 on ownership of phone and female access to phone in Nepal in revised manuscript (cleaned version).

Did participants receive any incentives or reimbursements for their participation in the study?

No. The participant did not receive any incentives or reimbursements for their participation in the study. Phone call was made by researcher in scheduled time and date so they need not need to pay for call.

Page 6, line 97. Please define 'regular clients' within the context of this study.

Thank you. Definition has been added. Please refer page 7 line #103-104 in revised manuscript (cleaned version).

Page 7, line 99. Countries around the world implemented slightly different lockdowns as a way to manage COVID-19 spread. Please provide a brief description about what a 'complete lockdown' meant in the Nepal context, especially in regard to health care access more generally. Also, How long did the lockdown go for? This information could be included in the introduction.

Thank you. The suggested information has been updated in lines #106-108 where we highlight about the duration of lockdown, definition of complete lockdown in study setting section.

Please include information about the contraceptive use of participants. Were all participants accessing family planning services when they were approached to participate in the study? Were they new or continuing clients? What type of contraception was being used (for example. % LARC vs short-term)? If available, please include data on the number of children participants had.

Thank you for the suggestion. This has been updated in Table-1 (General Information about study participants)

Data Collection

Page 8, table 1 – It is not clear how economic status or education level is determined and defined here. Please clarify.

The definition has been updated in the footnote of Table-1. Education status has been determined according to Government of Nepal classification and economic status is self-reported by respondents.

Please include information on who conducted the interviews.

Data was collected by female co-author. This has been updated in line #126 in revised manuscript.

Some of the codes provided in Table 2 are not easily understood, especially in relation to sub-themes. Please edit codes for clarity. Alternatively, could the definition of several sample codes be included for readers to better understand the coding and analysis process?

Thank you for the suggestion. Codes has been revised and operation definition has been added.

As a critical domain for qualitative inquiry, please include a brief section on research team reflexivity, including how interviewer characteristics could influence the participants response.

Team reflexivity has been added in discussion section. Please refer the line # 543-553 in revised manuscript (cleaned Version).

Has there been any dissemination of research findings to participants, or member-checking?

Yes. We checked with health service providers after completion of interviews in each district. This has been updated in line #133-135 in revised manuscript (cleaned version).

Data analysis

Please include more detail about the transcription and translation process. What language were the transcriptions made in? Who conducted the transcription process? At what stage was translation conducted, by who, and how was it quality controlled to ensure accuracy and meaning was maintained, especially for direct quotes?

This has been updated and revised in line #142-144 of revised manuscript (cleaned version). Data transcription was in Nepali by female co-author which was checked by PI and another author to ensure the quality.

Results

In general, more information and clarity is needed about what barriers existed before the pandemic. That is, were these barriers already present and were exacerbated or changed by the pandemic? Or did they not exist before?

Thank you for the suggestion. Some of these barriers exist before pandemic but the severity has been more during pandemic, which we have highlighted in overarching result in result section. Please refer the line #425-436 of revised manuscript (cleaned version).

Please be specific when referring to COVID-19 infection or disease. For example, page 13 line 151.

Thank you. Revised as Infection throughout the report.

Discussion:

Additional references would strengthen this section and overall paper.

Six additional references have been added to strengthen the paper.

Some repetition within the results section could also be removed.

Revised and some references has been removed.

Page 26, line 431. Present study includes women of reproductive age 15 – 49 years. Should be 18 – 49 years?

Thank You. Correction has been made. Please refer the line #464 in revised manuscript.
