# Supplementary material for: Barriers in accessing family planning services in Nepal during the COVID-19 pandemic: A qualitative study
Source: PLoS One. 2023 May 5;18(5):e0285248. doi: 10.1371/journal.pone.0285248 (PMC10162517; doi:10.1371/journal.pone.0285248)
Supplement: Attachment — Submitted filename: Reviewers comment.docx [file pone.0285248.s003.docx]

**Reviewer’s comment**

**Barriers in accessing family planning services in Nepal during the COVID-19 pandemic: A qualitative study**

Anil Sigdel, Anu Bista, Hardik Sapkota, Edwin van Teijlingen

This study informs me the barriers to access FP services during the Covid-19 in Nepal. It is good and informative to readers but the following points should be consider to improve this study before publications.

**Introduction**

It should be more elaborative to justify why this study should be conducted in Nepal. While the situation related with impacts of Covid-19 on FP services in general are well elaborative, the situation of Nepal is not described in introduction although some sentences are described in study settings. It will be more attractive to reader if the situation of Nepal would be mentioned before the study aim as why thus study should be done inevitably.

**Methods**

The reference for socio-ecological model should be added in line 145.

**Results**

According to the study aim, this study will identify the barriers. At the individual level, some of the quotes include both the barriers and facilitators or how to overcome the barriers to receive FP during the Covid-19. For examples, low self-confidence, inadequate knowledge, perceived importance. If so, it could be confused to readers even though these are informative. But, in the rest of the quotes, there are only barriers. I think it should be consistent with the objective and the whole structure.

Under the overarching lesson learnt, there are some sentences related with impact of Covid-19 on FP for global condition. I understand that it should be study specific overall acquired knowledge after conducting study. It should be considered again to be more relevant. Current sentences may be useful to be in discussion part.

**Discussion**

At line 502, it is not clear for “commodity based contraception”. Is it the same as modern contraception?

Also it was discussed about elaborately for fear of side effects while it was not mentioned in results or conclusion as important barriers.

In discussion, most the references used to compare were conducted before Covid-19. It would be more informative if there are compared with the study or literatures for specific Covid-19 context. And, some the barriers are due to Covid-19 and have not existed before Covid-19. Some are existing barriers for FP but more exaggerated due to Covid-19. This concept should be discussed thoroughly to be clear for readers.

In the discussion, I would like to suggest considering again to be more attractive. Now, the comparative findings are written for each barriers and then there no overall conceptualization of this study to convince to readers. It should also be connected with overall lesson learnt from results. Now it is difficult to follow what the authors want to highlight the key discussion points. Then, it should be connected with conclusion. The qualitative discussion should be like a good narrative storytelling to be more attractive to readers.

Strengths and limitations should be sub-tiled to clear for readers.
