# Supplementary material for: Barriers in accessing family planning services in Nepal during the COVID-19 pandemic: A qualitative study
Source: PLoS One. 2023 May 5;18(5):e0285248. doi: 10.1371/journal.pone.0285248 (PMC10162517; doi:10.1371/journal.pone.0285248)
Supplement: Attachment — Submitted filename: Rebuttal letter_15March2023.docx [file pone.0285248.s004.docx]

**Rebuttal letter**

We thank the academic editor and the two reviewers for their comments on our manuscript. Below is our response to each point raised by the academic editor and reviewers. We hope that we satisfyingly addressed them and that the manuscript will be now suited for publication.

Sincerely,

On behalf of all authors,

Anil Sigdel

**Reviewer #3**

**Abstract**
**As the study aim is to " explore barriers faced by women while seeking family planning services in Nepal during COVID-19 lockdown" line 92/93, the abstract should highlight more of the COVID 19 related findings and conclusions to bring light to the objective of the study.**
Thank you for the suggestions. Key highlights related to COVID-19 have been added in the abstract. Please review the revised abstract section.

**Introduction
Global COVID 19 pandemics have been addressed and the situation of the Nepal context and the lock down periods need to be spell out clearly with more emphasis on case load, fatality and vaccine coverage and most effected districts.
If there are previous studies looking into barriers in accessing family planning service in general, more information should be added as the focus is on family planning. Please also add the interruption of routine service data during the COVID 19.**

The information has been updated in lines 60-66 and some study context has also been provided in study setting (line 126-140) in revised manuscript.
**Methodology
For the qualitative methodology reporting, the COREQ (Consolidated Criteria for Reporting Qualitative Studies) is a gold atandard to follow and in this study there are some methodological issues that need to be addressed.
1. Research team composition and who were they and who did the interviews. Any bias in selection of particpants.**
Information related to research team composition and one conducting interview has been updated in line 142-144. Further, regarding bias has been updated in line 120-124 in revised manuscript.

**2. Line 111-112 the sample of the women in the study were selected from " three geographical areas (Mountain, Hills and Terai) of Nepal. These clinics are the outlet of one NGO working on SRH whose clinics are accredited by Family Welfare Division of Nepal and has stayed open during the lockdown". But in the study findings there were no differences mentioned between the geographical areas, please explain? Also is there a reason why you only chose the 5 district ( More covid cases??)**
Yes. The Prime reason is high number of COVID-19 cases in those five districts i.e. 39% of total COVID-19 cases Regarding outlet and representation, this NGO has countrywide clinic outlets and clinics in districts with high COVID-19 cases in each ecological region has been selected.

**3. You have chosen only women from outlet of one NGO on SRH clinic, and it is hard to draw conclusion to the whole Nepal without identifying the bias in qualitative sampling as well.**
Thank you. The bias in qualitative sampling has been highlighted in line 120-124 in revised manuscript.

4**. Were there any difficulties encountered during the telephone interviews? 30-40 min long talks on the phone and compensation for the phone charges needed to be spell out clearly. Please also mentioned reflexibility of the phone interviews within the team.**
Reflexibility on phone interview has been reflected in line 150-160 in revised manuscript. Please refer the revised manuscript.

**5. Please also reconsider the coding frame for more clarity of the themes and sub themes and the code** **definitions.**
Thank you. This has been considered. Please refer the updated code tree in revised manuscript.

**6. In line 115 saturation of the findings was reached but please mention how.**
This has been reflected in line 161-162. We noticed that the information was saturated because the last three interviews did not add any new information complementing the objectives and scope of the study.

**Results
Results session need to be readjusted to pay more attention to the COVID 19 related barriers rather than writing it in general.**
Thank you. This has been readjusted. Please refer to the revised manuscript.

**Discussion
Please do not repeat the results again in the discussing session.
Line 449-453: You talked about the low education and poverty for your result but in the comparison, it was with urban and rural. Please check and review.**
Thank you. Comparisons with other relevant literatures have been made in line 513-523 in revised manuscript.

**Your study includes women of reproductive age 19-49 and you often compare with the study of adolescent 15-24 which is hard to understand. Please find a suitable reference to compare the factors.**
Relevant literature has been updated. Please refer the line 512-523 in revised manuscript.

**Line 469-473: You have mentioned about the decision-making power of women with better education and living in nuclear family. However, you related to the mother-in-law as a decision maker in the comparison study which is confusing. Please aligned your stand and rephrase**.
This has been revised and compared with relevant literatures. Please refer to line 535-540 in revised manuscript.

**Line 486-495: I do understand that misinformation on FP may hinder women to seek services but how does that relate to COVID 19 is not clear. Does COVID 19 lockdown may have an indirect effect on information seeking for FP.**

Yes. The COVID-19 lockdown have indirect effect on information for FP as most of the information were confined with COVID-19 and people do not have access with right information to address the misconception regarding FP, which affect in up-take of FP services.

**Reviewer #4:**

**Introduction**

**It should be more elaborative to justify why this study should be conducted in Nepal. While the situation related with impacts of Covid-19 on FP services in general are well elaborative, the situation of Nepal is not described in introduction although some sentences are described in study settings. It will be more attractive to reader if the situation of Nepal would be mentioned before the study aim as why thus study should be done inevitably.**

Thank You for the suggestion. Additional information on situation of Nepal has been updated in introduction section in revised manuscript. Please refer the line 101-107.

**Methods**

**The reference for socio-ecological model should be added in line 145.**

Thank You. Updated.

**Results**

**According to the study aim, this study will identify the barriers. At the individual level, some of the quotes include both the barriers and facilitators or how to overcome the barriers to receive FP during the Covid-19. For examples, low self-confidence, inadequate knowledge, perceived importance. If so, it could be confused to readers even though these are informative. But, in the rest of the quotes, there are only barriers. I think it should be consistent with the objective and the whole structure.**

Thank you for the suggestion. This has been corrected.

**Under the overarching lesson learnt, there are some sentences related with impact of Covid-19 on FP for global condition. I understand that it should be study specific overall acquired knowledge after conducting study. It should be considered again to be more relevant. Current sentences may be useful to be in discussion part.**

Thank you for the suggestion. This has been considered in revised manuscript.

**Discussion**

**At line 502, it is not clear for “commodity based contraception”. Is it the same as modern contraception?**

Yes. This is a modern contraception. Community based contraception has been replaced with modern contraception in revised manuscript.

**Also it was discussed about elaborately for fear of side effects while it was not mentioned in results or conclusion as important barriers.**

This has been updated in result part mostly in social stigma section. Thank You

**In discussion, most the references used to compare were conducted before Covid-19. It would be more informative if there are compared with the study or literatures for specific Covid-19 context. And, some the barriers are due to Covid-19 and have not existed before Covid-19. Some are existing barriers for FP but more exaggerated due to Covid-19. This concept should be discussed thoroughly to be clear for readers.**

Thank you. Findings were compared with several studies conducted during COVID-19 and Ebola Crisis in revised discussion section. Further, different sections have been revised to show how COVID-19 further exaggerated the pre-existing barriers.

**In the discussion, I would like to suggest considering again to be more attractive. Now, the comparative findings are written for each barriers and then there no overall conceptualization of this study to convince to readers. It should also be connected with overall lesson learnt from results. Now it is difficult to follow what the authors want to highlight the key discussion points. Then, it should be connected with conclusion. The qualitative discussion should be like a good narrative storytelling to be more attractive to readers.**

Thank you. Key findings and highlights are discussed in discussion sections as suggested. Please refer to the revised discussion section of revised manuscript.

**Strengths and limitations should be sub-tiled to clear for readers.**

Thank You. This has been updated.
