# Supplementary material for: Barriers in accessing family planning services in Nepal during the COVID-19 pandemic: A qualitative study
Source: PLoS One. 2023 May 5;18(5):e0285248. doi: 10.1371/journal.pone.0285248 (PMC10162517; doi:10.1371/journal.pone.0285248)
Supplement: Attachment — Submitted filename: Rebuttal letter_16April2023.docx [file pone.0285248.s005.docx]

**Rebuttal letter**

We thank the academic editor and the two reviewers for their comments on our manuscript. Below is our response to each point raised by the reviewers. We hope that we satisfyingly addressed them and that the manuscript will be now suited for publication.

Sincerely,

On behalf of all authors,

Anil Sigdel

**Reviewer #4**

**Thank you for sharing the revised manuscripts which improves a lot and has been revised most of the comment. But one comment related with Overarching lesson learnt; "Under the overarching lesson learnt, there are some sentences related with impact of Covid-19 on FP for global condition. I understand that it should be study specific overall acquired knowledge after conducting study. It should be considered again to be more relevant. Current sentences may be useful to be in discussion part", was not revised yet. Actually, in the results part, the findings should be from your study/ information that you collected during interviews. It should not be from other literature/ reference. These references will be useful in discussion part. Here, it should be overall findings of your study should be conceptualized to convince to the readers.**

Thank you for the suggestion. This has been updated in the revised manuscript as, “The study found that along with the prevailing barriers in the uptake of FP, the global pandemic like COVID-19 have significant role in introducing/adding new additional barriers in uptake of FP services. The emergence of COVID-19 leads to movement restriction, financial hardship due to loss of job, un-acceptance of FP as essential health services, increased time at home with husband or parents, violation of privacy, increased waiting time at health facilities, limited outreach services by community health workers, delayed prepositioning of FP commodity and diversion of health workers to respond COVID-19 were noted as the additional barriers in uptake of FP services by women in low-and-middle-income countries like Nepal. This clearly implies that public health efforts, particularly SRH programs, should consider the additional challenges that occur as a result of the pandemic during the design and execution of public health programs.” Please refer to the overarching lesson learnt session line number 483-493.

Thank You. This has been updated.
